# Ezetimibe/simvastatin 10/40 mg versus atorvastatin 40 mg in high cardiovascular risk patients with primary hypercholesterolemia: a randomized, double-blind, active-controlled, multicenter study

**DOI:** 10.1186/1476-511X-11-18

**Published:** 2012-01-31

**Authors:** Paul Kah Hing Ling, Fernando Civeira, Andrei Gheorghe Dan, Mary E Hanson, Rachid Massaad, Celine Le Bailly De Tilleghem, Christopher Milardo, Joseph Triscari

**Affiliations:** 1 School of Medicine and Health Sciences, Monash University, 80100 Johor Bahru, Malaysia; 2 Hospital Universitario Miguel Servet, Zaragoza, Spain; 3 University Hospital Colentina of Bucharest, Bucharest, Romania; 4 Merck Sharp & Dohme Corp., a div. of Merck & Co., Inc., Whitehouse Station, NJ, USA; 5 Merck Sharp & Dohme Corp., Brussels, Belgium

**Keywords:** Ezetimibe/simvastatin, Atorvastatin, High cardiovascular risk, Primary hypercholesterolemia, Lipid-lowering

## Abstract

**Background:**

A considerable number of patients with severely elevated LDL-C do not achieve recommended treatment targets, despite treatment with statins. Adults at high cardiovascular risk with hypercholesterolemia and LDL-C ≥ 2.59 and ≤ 4.14 mmol/L (N = 250), pretreated with atorvastatin 20 mg were randomized to ezetimibe/simvastatin 10/40 mg or atorvastatin 40 mg for 6 weeks. The percent change in LDL-C and other lipids was assessed using a constrained longitudinal data analysis method with terms for treatment, time, time-by-treatment interaction, stratum, and time-by-stratum interaction. Percentage of subjects achieving LDL-C < 1.81 mmol/L, < 2.00 mmol/L, or < 2.59 mmol/L was assessed using a logistic regression model with terms for treatment and stratum. Tolerability was assessed.

**Results:**

Switching to ezetimibe/simvastatin resulted in significantly greater changes in LDL-C (-26.81% vs.-11.81%), total cholesterol (-15.97% vs.-7.73%), non-HDL-C (-22.50% vs.-10.88%), Apo B (-17.23% vs.-9.53%), and Apo A-I (2.56% vs.-2.69%) vs. doubling the atorvastatin dose (all *p *≤ 0.002), but not HDL-C, triglycerides, or hs-CRP. Significantly more subjects achieved LDL-C < 1.81 mmol/L (29% vs. 5%), < 2.00 mmol/L (38% vs. 9%) or < 2.59 mmol/L (69% vs. 41%) after switching to ezetimibe/simvastatin vs. doubling the atorvastatin dose (all *p *< 0.001). The overall safety profile appeared generally comparable between treatment groups.

**Conclusions:**

In high cardiovascular risk subjects with hypercholesterolemia already treated with atorvastatin 20 mg but not at LDL-C < 2.59 mmol/L, switching to combination ezetimibe/simvastatin 10/40 mg provided significantly greater LDL-C lowering and greater achievement of LDL-C targets compared with doubling the atorvastatin dose to 40 mg. Both treatments were generally well-tolerated.

**Trial registration:**

Registered at clinicaltrials.gov: NCT00782184

## Background

Despite substantial lipid-lowering with the use of statins, a considerable number of patients at high cardiovascular risk and/or with severely elevated low-density lipoprotein cholesterol (LDL-C) do not achieve the rigorous treatment targets recommended by European, Canadian and US guidelines [1-4]. LDL-C reductions beyond that achieved by currently used statin therapies are recommended for a considerable number of patients [4-7]. Clinical trial results have demonstrated that combining ezetimibe with a statin more effectively lowers LDL-C vs. treatment with either of the individual components alone [7,8]. In addition, combining ezetimibe with simvastatin has been shown to provide greater cholesterol lowering vs. doubling the statin dose in patients with hypercholesterolemia [9], in those at high risk for coronary heart disease (CHD) [10], and in patients with either diabetes [11,12] or metabolic syndrome [13].

Previous studies included other dose ranges, populations with type 2 diabetes or metabolic syndrome, or did not include target attainment as a primary or secondary endpoint. The objective of this study was to assess the efficacy and tolerability profile of switching to ezetimibe/simvastatin 10/40 mg compared with doubling the baseline atorvastatin dose to 40 mg in high cardiovascular risk subjects with primary hypercholesterolemia and not at LDL-C goal of < 2.59 mmol/L with atorvastatin 20 mg treatment at baseline. This trial assessed percent change from baseline in LDL-C as the primary endpoint and attainment of LDL-C targets as the secondary endpoints using ezetimibe added to simvastatin 40 mg compared with atorvastatin 40 mg in a population of subjects at high risk for CHD. Other secondary endpoints included the percent change from baseline in other lipids, lipid ratios, and high-sensitivity C-reactive protein (hs-CRP) after the 6-week treatment period.

## Methods

This was a randomized, double-blind, active-controlled, 2-arm, multicenter study conducted at 60 sites (11 in the US; 7 in Malaysia; 6 each in Hungary, Poland, and Spain; 5 in Romania; 4 each in Costa Rica, Guatemala, Latvia, and Peru; 2 in Estonia; and 1 in Israel) from November 2008 to September 2010. The protocol (Protocol 134) and amendments were reviewed and approved by an institutional review board. The study was conducted in conformance with applicable country or local requirements regarding ethical committee review, informed consent, and other statutes or regulations regarding the protection of the rights and welfare of human subjects participating in biomedical research. All subjects signed informed consent prior to any study procedures being performed.

### Subjects

Eligible subjects were adults 18-79 years of age at high risk for CHD with primary hypercholesterolemia. For 6 weeks prior to screening, subjects eligible for the run-in period were currently taking either atorvastatin 20 mg or another lipid-lowering therapy with potency ≤ atorvastatin 20 mg, or were naïve to lipid-lowering therapy (naïve was defined as not being treated with a statin and/or ezetimibe). During the 5-week run-in period, subjects received open label atorvastatin 20 mg and lifestyle, diet counseling, and treatment compliance recommendations. All subjects must have been at high risk as defined by the National Cholesterol Education Program Adult Treatment Panel (NCEP ATP) III, which included subjects with CHD, having a CHD risk equivalent medical condition, and those who had 2+ risk factors that confer a 10-year risk for CHD > 20% as determined by the Framingham calculation [3]. Laboratory entry criteria included LDL-C ≥ 100 mg/dL (2.59 mmol/L) and ≤ 160 mg/dL (4.14 mmol/L) at baseline (Visit 2, which is a treated baseline following the active treatment run-in period), triglyceride level ≤ 4.0 mmol/L (≤ 350 mg/dL), liver transaminases (alanine aminotransferase [ALT] and aspartate aminotransferase [AST]) ≤ 2.0 × upper limit of normal (ULN) with no active liver disease, and creatine kinase (CK) levels ≤ 3x ULN. Subjects were excluded from participating if they were taking simvastatin 80 mg, atorvastatin 40 or 80 mg, or rosuvastatin 10, 20, or 40 mg, or were taking other prescription and/or over-the-counter-drugs with the potential for significant lipid effects (other than study drug) or with potential drug interactions with the statins. Females who were pregnant or lactating were also excluded.

### Blinding & randomization

The Clinical Biostatistics department of the study sponsor generated the randomized allocation schedule for study treatment assignment. At the end of the run-in period, eligible subjects were randomized in a 1:1 ratio to atorvastatin 40 mg or ezetimibe/simvastatin 10/40 mg. To achieve balance across treatment groups, subjects were stratified by their baseline LDL-C values at randomization (taken at Visit 3): Stratum 1: ≥ 2.59 mmol/L (100 mg/dL) and < 3.36 mmol/L (130 mg/dL) and Stratum 2: ≥ 3.36 mmol/L (130 mg/dL) and ≤ 4.14 mmol/L (160 mg/dL). Subjects received open label run-in bottles containing atorvastatin 20 mg. Treatment was supplied in blinded kits, each containing 2 bottles labeled A and B. Subjects were instructed to take one tablet daily from each of the bottles provided. The final database was not unblinded until medical/scientific review was performed, protocol violators were identified, and data had been declared final and complete.

### Efficacy measures

LDL-C was calculated using the Friedewald method when triglycerides were < 4.52 mmol/L (400 mg/dL). The beta quantification ultracentrifugation method was used if triglycerides reached ≥ 4.52 mmol/L (400 mg/dL). Total cholesterol (TC), high-density lipoprotein cholesterol (HDL-C), hs-CRP, apolipoprotein (Apo) A-I, Apo B, and triglyceride fasting plasma levels were determined by a central laboratory. Non-HDL-C, LDL-C/HDL-C ratio, TC/HDL-C ratio, non-HDL-C/HDL-C ratio, and Apo B/Apo A-I ratio were calculated using the laboratory measurements.

### Safety measures

Clinical evaluation included physical examinations, vital signs (blood pressure, weight, height, pulse rate), and laboratory safety tests. Clinical safety evaluations, including serious adverse events (AEs) and discontinuations due to clinical and/or laboratory AEs, were performed at Visits 2, 3, 4, and 5. Laboratory safety test evaluations were performed at Visits 1, 3, and 5 during the study.

### Statistical methods

#### Sample size

Approximately 240 patients were expected to be randomized to ensure that 220 evaluable subjects would be included in the analysis of the full analysis set (FAS) population (110 per treatment group). With this enrollment, the study was assumed to have 95% power to detect a treatment difference of 10% in percent change from baseline between ezetimibe/simvastatin 10/40 mg and atorvastatin 40 mg assuming a standard deviation (SD) of 20% (at significance level = 0.05, 2-sided).

#### Statistical analysis

Primary and secondary efficacy data were analyzed based on the FAS population, which included all randomized subjects who had a baseline measurement and received at least 1 dose of study medication. For the primary and secondary efficacy endpoints involving percent change from baseline (except triglycerides and hs-CRP), between-group differences were analyzed using a constrained longitudinal data analysis (LDA) model, including both baseline and post-baseline percent change from baseline measurements as the response variable, and with terms for treatment, time, the interaction of time-by-treatment, stratum, and the interaction of time-by-stratum with a restriction of the same baseline mean across treatment groups (due to randomization). Due to the non-normal distribution associated with percent changes from baseline in triglycerides and hs-CRP, data were log-transformed. The percentage of subjects reaching LDL-C < 1.81 mmol/L (70 mg/dL), < 2.00 mmol/L (77 mg/dL) and < 2.59 mmol/L (100 mg/dL), respectively, was analyzed using a logistic regression model with terms for treatment and stratum.

There were 1 primary and 3 secondary efficacy hypotheses. Since there was only 1 primary efficacy endpoint and only 1 primary treatment comparison, no adjustment for multiplicity was applied for the primary hypothesis and the treatment comparison was performed at significance level 0.05. Analysis of the secondary variables related to secondary hypotheses was performed if the primary variable was significant at level 0.05. A step-down ordered testing procedure, which keeps the overall Type I error at level 0.05 if each test is performed at level 0.05, was performed for the key secondary variables. Specifically, the primary comparison of ezetimibe/simvastatin (10/40 mg) versus atorvastatin 40 mg in percent change from baseline in LDL-C was performed first. The tests of the secondary hypotheses were performed in the following order if the primary endpoint was significant at level 0.05: (1) percentage of subjects reaching LDL-C goal of < 1.81 mmol/L (70 mg/dL), (2) percentage of subjects reaching LDL-C goal of < 2.00 mmol/L (77 mg/dL), (3) percentage of subjects reaching LDL-C goal of < 2.59 mmol/L (100 mg/dL). The ordered testing procedure was to stop at any step where statistical significance was not achieved.

### Safety data

The All Patients as Treated (APaT) population, which consisted of all randomized subjects who received at least one dose of study treatment, was used for the analysis of safety data. Inferential testing provided statistical significance levels for between-group comparisons on the following safety parameters: gastrointestinal-related AEs, gallbladder-related AEs, allergic reaction or rash AEs, hepatitis-related AEs, elevations in ALT/AST ≥ 3× ULN, elevations in CK ≥ 10× ULN, elevations in CK ≥ 10× ULN with muscle symptoms and elevations in CK ≥ 10× ULN with drug-related muscle symptoms. Ninety-five percent confidence intervals (CIs) for treatment differences in proportions using Miettinen and Nurminen were provided for safety parameters including: 1 or more AEs, drug-related AEs, serious AEs, discontinuations due to an AE, AEs and predefined limits of change in laboratory safety parameters with incidence occurring in at least 4 subjects within at least 1 of the treatment groups. Descriptive statistics were used for all other AEs and predefined limits of change

## Results

**Figure 1:**
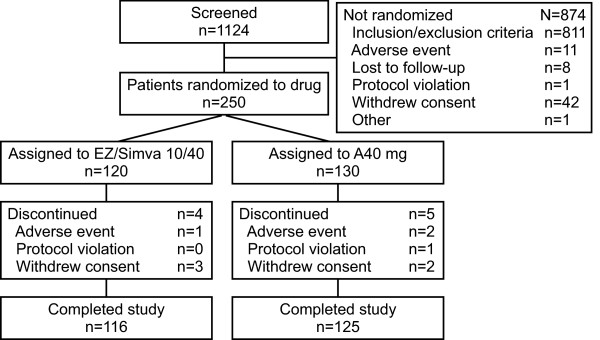
**Patient flow through the study**.

The flow of subjects through the study is summarized in Figure 1. Of the 1124 subjects screened, 874 subjects were excluded, leaving 250 subjects to be randomized. There were 120 subjects randomized to the ezetimibe/simvastatin group and 130 to the atorvastatin group. There were a total of 9 discontinuations, which included 4 from the ezetimibe/simvastatin group and 5 from the atorvastatin group.

Baseline demographics and clinical characteristics were generally similar between treatment groups (Table 1). The majority of subjects were white (72%) and the mean age was 59.3 years (standard deviation [SD] = 9.2). Baseline lipids were generally similar between groups, with baseline mean LDL-C = 3.12 mmol/L (SD = 0.42) (120.44 mg/dL [SD = 16.04])

**Table 1:** Baseline demographics and clinical characteristics

	EZ/Simva 10/40 mg n (%)	Atorvastatin 40 mg n (%)	Total
	N = 120	N = 130	N = 250
Male	63 (52.5)	65 (50.0)	128 (51.2)
Mean age (SD)	58.9 (10.0)	59.7 (8.4)	59.3 (9.2)
Age ≥ 65 years	36 (30.0)	36 (27.7)	72 (28.8)
Race			
White	89 (74.2)	90 (69.2)	179 (71.6)
Asian	21 (17.5)	24 (18.5)	45 (18)
Multi-Racial	10 (8.3)	14 (10.8)	24 (9.6)
American Indian or Alaska Native	0	1 (0.8)	1 (0.4)
Black or African American	0	1 (0.8)	1 (0.4)
Ethnicity--Hispanic or Latino	25 (20.8)	29 (22.3)	54 (21.6)
Baseline LDL-C Stratum			
Stratum 1: ≥ 2.59 mmol/L (100 mg/dL) to < 3.36 mmol/L (130 mg/dL)	81 (67.5)	97 (74.6)	178 (71.2)
Stratum 2: ≥ 3.36 mmol/L (130 mg/dL) to < 4.14 mmol/L (160 mg/dL)	39 (32.5)	33 (25.4)	72 (28.8)
BMI ≥ 30 kg/m^2^	43 (37.1)	41 (32.3)	84 (34.6)
History of Diabetes	42 (35.0)	45 (34.6)	87 (34.8)

Clinical Characteristics, mean (SD)			

LDL-C, mmol/L mg/dL	3.16 (0.43) 122.00 (16.5)	3.08 (0.40) 119.01 (15.52)	3.12 (0.42) 120.44 (16.04)
Total Cholesterol, mmol/L mg/dL	5.25 (0.55) 202.54 (21.1)	5.14 (0.60) 198.60 (23.11)	5.19 (0.59) 200.49 (22.21)
Triglycerides*, mmol/L mg/dL	1.53 (0.76) 135.00 (66.98)	1.49 (0.76) 131.50 (66.98)	1.49 (0.76) 132.00 (66.98)
HDL-C, mmol/L mg/dL	1.33 (0.33) 51.54 (12.87)	1.33 (0.35) 51.18 (13.28)	1.33 (0.34) 51.36 (13.06)
non-HDL-C, mmol/L mg/dL	3.91 (0.51) 151.00 (19.56)	3.82 (0.51) 147.42 (19.68)	3.86 (0.51) 149.14 (19.67)
Apo B, g/L (mg/dL)	1.20 (0.16) 119.87 (16.06)	1.17 (0.16) 117.32 (15.83)	1.19 (0.16) 118.54 (15.96)
Apo A-I, g/L (mg/dL)	1.15 (0.25) 155.15 (24.78)	1.58 (0.28) 157.69 (28.28)	1.60 (0.27) 156.47 (26.63)
LDL-C/HDL-C	2.50 (0.64)	2.46 (0.65)	2.48 (0.64)
Total C/HDL-C	4.12 (0.89)	4.07 (0.91)	4.09 (0.9)
non-HDL-C/HDL-C	3.12 (0.89)	3.07 (0.91)	3.09 (0.90)
Apo B/Apo A-I	0.80 (0.17)	0.77 (0.18)	0.78 (0.18)
hs-CRP*, mg/L	2.15 (2.7)	2.10 (2.7)	2.10 (2.7)

Apo, apolipoprotein; BMI, body mass index; EZ, ezetimibe; HDL-C, high-density lipoprotein cholesterol; hs-CRP, high-sensitivity C-reactive protein; LDL-C, low-density lipoprotein cholesterol; SD, standard deviation; simva, simvastatin

*Values are median and robust SD, calculated for triglycerides and hs-CRP as: interquartile range (IQR)/1.075, where IQR = 3rd quartile minus 1st quartile

**Figure 2:**
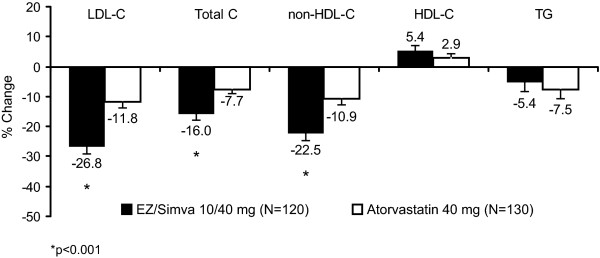
**Percent change from baseline in lipid levels after 6 weeks of treatment (Full Analysis Set population)**.

**Figure 3:**
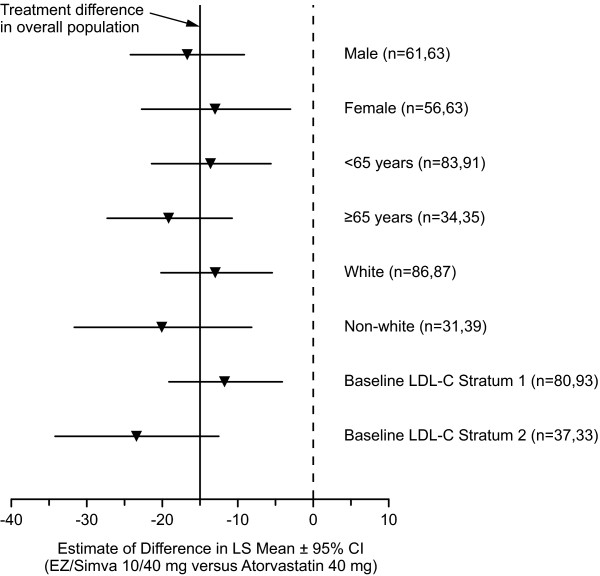
**Estimate of Treatment Difference in percent Change from Baseline in LDL-C (LS Mean ± 95% CI) Within Each Level of Specific Subgroup at Study Endpoint After 6 Weeks of Treatment (Full Analysis Set Population)**.

**Figure 4:**
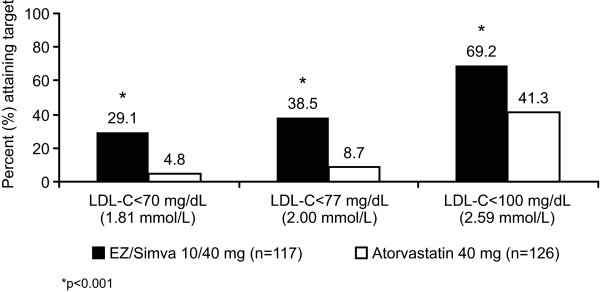
**Percentage of subjects attaining prespecified LDL-C targets after 6 weeks of treatment (Full Analysis Set population)**.

After 6 weeks of treatment, ezetimibe/simvastatin 10/40 mg resulted in significantly greater reductions from treated baseline in LDL-C levels compared with doubling the dose of atorvastatin to 40 mg (-26.8% vs -11.8%; *p *< 0.001 [Figure 2]). The estimated between-treatment difference was -15.0% (95% confidence interval [CI]: -21.15, -8.84) in favor of ezetimibe/simvastatin 10/40 mg therapy. The greater efficacy of the ezetimibe/simvastatin 10/40 mg treatment in reducing LDL-C was consistent across prespecified subgroups (Figure 3). Significantly more subjects attained all prespecified LDL-C targets with ezetimibe/simvastatin 10/40 mg vs atorvastatin 40 mg after 6 weeks of treatment (all *p *< 0.001; Figure 4). The percentage of subjects reaching LDL-C < 1.81 mmol/L (70 mg/dL), < 2.00 mmol/L (77 mg/dL) and < 2.59 mmol/L (100 mg/dL), respectively, was 29.1% vs 4.8% in the ezetimibe/simvastatin 10/40 mg group vs the atorvastatin 40 mg group; 38.5% vs. 8.7% in the ezetimibe/simvastatin 10/40 mg group vs the atorvastatin 40 mg group; and 69.2% vs 41.3% in the ezetimibe/simvastatin 10/40 mg group vs. the atorvastatin 40 mg group.

In addition to greater reductions in LDL-C, ezetimibe/simvastatin treatment resulted in significantly greater reductions in TC (*p *< 0.001), non-HDL-C (*p *< 0.001), Apo B (*p *= 0.002), Apo A-I (*p *< 0.001), and all lipid ratios (all *p *< 0.001; Table 2). There were no significant differences between treatments in change from baseline in triglycerides (*p *= 0.593), HDL-C (*p *= 0.211), or hs-CRP (*p *= 0.785; Table 2).

**Table 2:** Percent change from baseline in lipids, lipoproteins, lipid and apolipoprotein ratios and hs-CRP after 6 weeks of treatment (Full Analysis Set population)

	Final absolute lipid value		LS mean% change			
	**EZ/Simva** **10/40 mg**	**Atorvastatin** **40 mg**	**EZ/Simva 10/40 mg**	**Atorvastatin 40 mg**	Between-treatment difference*	***p*-value**

LDL-C, mmol/L (mg/dL)	2.32 (89.75)	2.79 (107.81)	-26.81	-11.81	-15.00	< 0.001
Total cholesterol, mmol/L (mg/dL)	4.45 (171.67)	4.84 (186.83)	-15.97	-7.73	-8.24	< 0.001
Triglycerides^†‡^, mmol/L (mg/dL)	1.40 (124.00)	1.34 (119.00)	-5.41	-7.54	2.13	0.593
HDL-C, mmol/L (mg/dL)	1.39 (53.54)	1.35 (51.94)	5.37	2.89	2.48	0.211
Non-HDL-C, mmol/L (mg/dL)	3.06 (118.13)	3.49 (134.89)	-20. 50	-10.88	-11.62	< 0.001
Apo B, g/L (mg/dL)	1.00 (99.72)	1.08 (108.52)	-17.23	-9.53	-7.69	0.002
Apo A-I, g/L (mg/dL)	1.58 (158.71)	1.51 (151.14)	2.56	-2.69	5.25	< 0.001
LDL-C/HDL-C	1.77	2.20	-28.77	-12.66	-16.1	< 0.001
Total C/HDL-C	3.35	3.77	-18.63	-8.60	-10.02	< 0.001
non-HDL-C/HDL-C	2.35	2.77	-24.41	-11.20	-13.21	< 0.001
Apo B/Apo A-I	0.65	0.73	-18.59	-5.67	-12.91	< 0.001
hs-CRP^†‡^, mg/L	1.70	1.80	-6.18	-8.86	2.68	0.785

Apo, apolipoprotein; BMI, body mass index; EZ, ezetimibe; HDL-C, high-density lipoprotein cholesterol; hs-CRP, high-sensitivity C-reactive protein; LDL-C, low-density lipoprotein cholesterol; LS, least squares; simva, simvastatin

*EZ/Simva (10/40 mg) - Atorvastatin 40 mg based on the least squares mean from a constrained LDA model with terms for treatment, time, time-by-treatment interaction, stratum and time-by-stratum interactions

^†^Final absolute values are medians

^‡^Data were transformed by the natural logarithm; the difference in least squares means was based on the difference in the back transformed model-based least squares means and the associated confidence interval was calculated using the delta method. Geometric mean percent changes from baseline levels were calculated based on back-transformation via exponentiation of the model-based least squares means and expressed as (geometric mean - 1) multiplied by 100

The incidence of clinical and laboratory adverse experiences was generally similar between treatment groups (Table 3). Discontinuations due to AEs were infrequent and similar between treatments (2 in the atorvastatin group and 0 in the ezetimibe/simvastatin group). There were no discontinuations due to serious AEs and 2 discontinuations due to drug-related AEs, both in the atorvastatin 40 mg group. There were 2 serious AEs reported (1 in each group) and neither was considered related to study drug. There was no significant difference between ezetimibe/simvastatin and atorvastatin in the percentage of subjects with gastrointestinal-related, allergic reactions or rash-related AEs, or hepatitis-related AEs. No gallbladder-related AEs occurred in the study and there were no subjects with single or consecutive elevations in ALT ≥ 3× ULN or AST ≥ 3× ULN or with elevation in CK ≥ 10× ULN. There were no deaths reported.

**Table 3:** Summary of safety data (All Patients as Treated population)

	EZ/Simva 10/40 mg N = 119 n (%)	Atorvastatin 40 mg N = 130 n (%)
≥ 1AE	11 (9.2)	18 (13.8)
Drug-related* AEs	2 (1.7)	3 (2.3)
Serious AEs	1 (0.8)	1 (0.8)
Discontinuations^†^		
due to an AE	0	2 (1.5)
due to a drug-related AEs	0	2 (1.5)
Gallbladder-related AEs	0	0
Gastrointestinal-related AEs	1 (0.8)	5 (3.8)
Hepatitis-related AEs	1 (0.8)	0
Allergic reaction or rash	0	2 (1.5)
ALT ≥ 3× ULN or AST ≥ 3× ULN	0	0
Creatinine kinase ≥ 10× ULN	0	0

*Determined by the investigator to be related to study drug

^†^Study medication withdrawn

AE, adverse event; ALT, alanine aminotransferase; AST, aspartate aminotransferase; EZ, ezetimibe; simva, simvastatin; ULN, upper limit of normal

## Discussion

These results demonstrated that compared with doubling the dose of atorvastatin to 40 mg, switching to the combination ezetimibe/simvastatin 10/40 mg resulted in significantly greater reductions in LDL-C levels and greater achievement of prespecified LDL-C targets in subjects with primary hypercholesterolemia at high risk for CHD who had not achieved LDL-C < 2.59 mmol/L (100 mg/dL) with atorvastatin 20 mg. In addition, TC, non-HDL-C, Apo B, and all lipid ratios were reduced while Apo A-I was increased significantly more with combination treatment compared with atorvastatin monotherapy. The safety and tolerability profiles were generally similar for both treatment groups.

It has been established that adding ezetimibe to statin monotherapy results in greater reductions in LDL-C and other lipids compared with statin monotherapy, even when existing statin therapy is doubled [14]. The mean LDL-C reduction observed in the current trial was consistent with expectations for the combination treatment, which is an approximately 15% greater reduction vs doubling the statin. In previous studies, mean reductions in LDL-C of at least 15% beyond that seen with a comparable or greater atorvastatin dose has been observed with the addition of ezetimibe to a statin (i.e., atorvastatin 20 mg plus ezetimibe 10 mg vs atorvastatin 40 mg [15]; atorvastatin 40 mg plus ezetimibe 10 mg vs atorvastatin 80 mg [16]; or ezetimibe/simvastatin 10/40 mg vs simvastatin 40 mg [7]). In other studies, although still significantly greater, the magnitude of difference between treatments has been smaller. For example, in a study of subjects with metabolic syndrome, the mean reduction in LDL-C was 8% greater with ezetimibe/simvastatin 10/40 mg compared with atorvastatin 40 mg [13]. And in the Vytorin Versus Atorvastatin (VYVA) study, which assessed subjects with hypercholesterolemia and established CHD or CHD risk equivalent, ezetimibe/simvastatin 10/40 mg resulted in a 9% greater decrease in LDL-C compared with atorvastatin 40 mg [17].

This variation between studies could be due to differences in study design or subject population; however, these differences may be mathematical in nature. The current study, as well as the others that show the larger magnitude of difference between treatments, included subjects with a 5- or 6-week treatment run-in period (or a treated baseline), while the studies with the smaller magnitudes of difference between treatments had wash-out periods or enrolled subjects who were naïve to lipid-lowering treatment. In the former studies (without washout) the baseline LDL-C level values were determined after statin therapy and the percent reductions may reflect the percent additional reduction using a larger denominator. In contrast, subjects with baseline LDL-C level values prior to statin therapy would potentially describe a smaller LDL-C reduction, although the actual LDL-C lowering was actually the same. Interestingly, in a *post hoc *analysis that compared LDL-C lowering with ezetimibe/simvastatin 10/20 mg with rosuvastatin 10 mg in subjects stratified by statin potency/dose prior to randomization, the subjects taking the higher potency/dose statins prior to switch experienced a greater magnitude of effect when switching to ezetimibe/simvastatin compared with rosuvastatin monotherapy; and more subjects achieved suggested therapeutic targets [18].

The results of this trial are also consistent with those of other trials in which significantly more subjects who switched from atorvastatin monotherapy (on which suggested LDL-C targets were not achieved) to combination ezetimibe/simvastatin attained individual LDL-C targets as recommended by the NCEP ATP III [3,9,10,19]. Results of trials with placebo run-in were also consistent with the current study and this was true even in populations of metabolic syndrome subjects [13,20]. In one study of subjects with type 2 diabetes, numerically more subjects achieved LDL-C < 2.59 mmol/L with ezetimibe/simvastatin 10/40 mg vs atorvastatin 40 mg; however, the difference was not significant between treatment groups (93.4% vs 89%, *p *< 0.07) [12].

## Conclusion

Both treatment regimens were generally well-tolerated with very few discontinuations. There were no reports of elevations in liver enzymes, which was consistent with expectations based on the results of previous trials. These results should be interpreted with caution since the study was not powered to detect very rare AEs. In conclusion, switching to combination ezetimibe/simvastatin 10/40 mg provides a generally well-tolerated lipid-lowering therapy for high cardiovascular risk subjects with hypercholesterolemia previously treated with atorvastatin 20 mg but not attaining LDL-C < 2.59 mmol/L.

## Abbreviations

AE: Adverse event; ALT: Alanine aminotransferase; AST: Aspartate aminotransferase; APaT: All patients as treated; Apo: Apolipoprotein; CHD: Coronary heart disease; FAS: Full analysis set; HDL-C: High-density lipoprotein cholesterol; hs-CRP: High-sensitivity C-reactive protein; LDA: Longitudinal data analysis; LDL-C: Low-density lipoprotein cholesterol; NCEP ATP III: National Cholesterol Education Program Adult Treatment Panel III; SD: Standard deviation.

## Competing interests

Dr. Ling reports no conflicts of interest regarding this manuscript. Dr. Civeira reports receiving grant money from Merck to him and his institution for the conduct of this study; he also has served as a consultant to Merck and on speakers' bureaus for AstraZeneca, Merck, and Pfizer. Dr. Dan reports receiving payments to himself and his institution for the conduct of this study. Drs. Hanson and Triscari and Ms. De Tilleghem are Merck employees and own stock and/or stock options in the company. Dr. Massaad is a MSD Europe employee. Mr. Milardo was a Merck employee from February 2003 through March 2011.

## Authors' contributions

All authors meet the criteria for authorship stated in the Uniform Requirements for Manuscripts Submitted to Biomedical Journals. Specifically, all authors made substantial contributions to the conception and design of the study and manuscript, contributed to the drafting of the article or revising it critically for important intellectual content, and all gave final approval of the version to be published.
